# Supplementary material for: Effects of Multi-Ingredient Preworkout Supplementation across a Five-Day Resistance and Endurance Training Microcycle in Middle-Aged Adults
Source: Nutrients. 2020 Dec 9;12(12):3778. doi: 10.3390/nu12123778 (PMC7764411; doi:10.3390/nu12123778)
Supplement: Supplementary file 1 [file nutrients-12-03778-s001.pdf]

Table S1. Mean (M) ± standard deviation (SD) and 95% CI of the differences measured after the three resistance training sessions for the tensiomyography variables determined in the two assessed conditions.

Muscles	Conditions	PREW (n=14)			CHO (n=14)			ANOVA Repeated Measures (3 workouts x 2 supplements)
	Variables	RT 1	RT 2	RT 3	RT 1	RT 2	RT 3	
Anterior Deltoids	Vc ($m{\cdot}s^{-1}$)	0.002 ± 0.04 [-0.02, 0.03]	0.01 ± 0.1 [-0.02, 0.04]	0.02 ± 0.1 [-0.02, 0.05]	-0.001 ± 0.1 [-0.03, 0.03]	0.01 ± 0.1 [-0.03, 0.05]	0.00 ± 0.1 [-0.04, 0.05]	Workout: F(2,24)=0.970; p=0.392; η^2 = 0.009<br>Supplement: F(1,13)=0.393; p=0.542; η^2 = 0.003<br>Workout x Supplement: F(2,24)=0.797; p=0.461; η^2 = 0.004
	Dm (mm)	-0.08 ± 1.7 [-1.11, 0.95]	0.53 ± 2.1 [-0.75, 1.82]	0.58 ± 2.2 [-0.72, 1.89]	-0.18 ± 2.1 [-1.47, 1.10]	0.53 ± 2.6 [-1.05, 2.11]	-0.02 ± 2.8 [-1.72, 1.68]	Workout: F(2,24)=1.325; p=0.283; η^2 = 0.014<br>Supplement: F(1,13)=0.447; p=0.516; η^2 = 0.003<br>Workout x Supplement: F(2,24)=0.457; p=0.638; η^2 = 0.003
	Tc (ms)	0.04 ± 2.4 [-1.42,1.49]	0.52 ± 2.0 [-0.69, 1.73]	0.46 ± 2.4 [-0.96, 1.87]	-0.03 ± 2.1 [-1.30, 1.24]	0.31 ± 2.3 [-1.09, 1.71]	-0.08 ± 1.8 [-1.18, 1.02]	Workout: F(2,26)=0.490; p=0.618; η^2 =0.006<br>Supplement: F(1,13)=0.623; p=0.444; η^2 =0.004<br>Workout x Supplement: F(2,26)=0.199; p=0.821; η^2 =0.002
Biceps Femoris Long Head	Vc ($m{\cdot}s^{-1}$)	0.001 ± 0.02 [-0.01, 0.02]	0.01 ± 0.02 [-0.01, 0.02]	0.02 ± 0.02 [0, 0.03]	-0.004 ± 0.02 [-0.02, 0.01]	0.001 ± 0.03 [-0.02, 0.02]	0.002 ± 0.03 [-0.02, 0.02]	Workout: F(2,24)=1.141; p=0.336; η^2 = 0.008<br>Supplement: F(1,12)=0.001; p=0.976; η^2 = 0.001<br>Workout x Supplement: F(2,24)=0.503; p=0.611; η^2 = 0.001
	Dm (mm)	-0.12 ± 1.3 [-0.94, 0.7]	0.23 ± 1.43 [-0.67, 1.13]	0.32 ± 1.46 [-0.60, 1.24]	-0.28 ± 1.20 [-1.03, 0.47]	-0.28 ± 1.70 [-1.35, 0.79]	-0.20 ± 1.75 [-1.29, 0.91]	Workout: F(2,24)=0.272; p=0.765; η^2 = 0.001<br>Supplement: F(1,12)=0.026; p=0.874; η^2 = 0.000<br>Workout x Supplement: F(2,24)=0.040; p=0.961; η^2 = 0.000
	Tc (ms)	-1.20 ± 10.78 [-7.81, 5.42]	-1.46 ± 8.94 [-6.77, 3.85]	-3.05 ± 9.49 [-8.56, 2.47]	-0.20 ± 8.4 [-5.16, 4.76]	-3.6 ± 8.85 [-8.43, 1.23]	-2.83 ± 7.98 [-7.33, 1.67]	Workout: F(2,24)=0.397; p=0.677; η^2 = 0.003<br>Supplement: F(1,12)=0.266; p=0.615; η^2 = 0.001<br>Workout x Supplement: F(2,24)= 0.474; p=0.628; η^2 = 0.038
Vastus Medialis	Vc ($m{\cdot}s^{-1}$)	-0.004 ± 0.02 [-0.02, 0.01]	0.001 ± 0.03 [-0.02, 0.02]	-0.02 ± 0.02 [-0.02, 0.01]	-0.006 ± 0.02 [-0.02, 0.01]	-0.002 ± 0.02 [-0.02, 0.01]	-0.01 ± 0.02 [-0.02, 0.00]	Workout: F(1,12)=1.561; p=0.231; η^2 = 0.019<br>Supplement: F(1,12)=0.992; p=0.339; η^2 = 0.018<br>Workout x Supplement: F(2,24)=1.052; p=0.365; η^2 = 0.009
	Dm (mm)	-7.14 ± 1.5 [-1.64, 0.21]	-0.36 ± 1.8 [-1.46, 0.74]	-0.63 ± 1.4 [-1.44, 0.18]	-0.82 ± 1.2 [-1.54, -0.1]	-0.59 ± 1.16 [-1.3, 0.12]	-0.93 ± 1.23 [-1.68, -0.17]	Workout: F(2,24)=1.779; p=0.190; η^2 = 0.013<br>Supplement: F(1,12)=1.364; p=0.265; η^2 = 0.013<br>Workout x Supplement: F(2,24)=0.542; p=0.589; η^2 = 0.004
	Tc (ms)	-3.37 ± 12.5 [-11.14, 4.4]	-2.57 ± 13.3 [-10.88, 5.74]	-3.9 ± 12.2 [-11.28, 3.48]	-3.62 ± 12.2 [-11.19, 3.95]	-3.38 ± 12.4 [-11.17, 4.42]	-3.37 ± 11.3 [-10.44, 3.7]	Workout: F(2,24)=0.355; p=0.705; η^2 = 0.001<br>Supplement: F(1,12)=1.247; p=0.286; η^2 = 0.002<br>Workout x Supplement: F(2,24)=1.176; p=0.326; η^2 = 0.002

Notes: All values are adjusted using sex as covariate. All P>0.05

Table S2. Responses to the questionnaire of sensitive feelings

Question	Condition	RT 1	RT 2	RT 3	Average
My energy level is	PREW	3.7 ± 1	3.5 ± 1	4.0 ± 1	3.7 ± 1
	CHO	3.7 ± 1	3.9 ± 1	3.7 ± 1	3.8 ± 1
My fatigue level is	PREW	1.9 ± 1	3.5 ± 1	2.0 ± 1	2.1 ± 1
	CHO	1.9 ± 1	3.9 ± 1	1.9 ± 1	1.9 ± 1
My feeling of alertness is	PREW	3.7 ± 1	3.6 ± 1	3.8 ± 1	3.7 ± 1
	CHO	3.6 ± 1	4.0 ± 1	3.9 ± 1	3.9 ± 1
My feeling of focus for task is:	PREW	3.8 ± 1	3.9 ± 1	4.1 ± 1	3.9 ± 1
	CHO	3.6 ± 1	4.0 ± 1	4.0 ± 1	3.9 ± 1

PREW = Preworkout supplement; CHO = Carbohydrate supplement. All data are reported as Mean ± SD.
